# Clinical impact of a *Clostridioides* (*Clostridium*) *difficile* bedside infectious disease stewardship intervention

**DOI:** 10.1093/jacamr/dlaa037

**Published:** 2020-08-11

**Authors:** María Olmedo, Maricela Valerio, Elena Reigadas, Mercedes Marín, Luis Alcalá, Patricia Muñoz, Emilio Bouza

**Affiliations:** 1 Department of Clinical Microbiology and Infectious Diseases, Hospital General Universitario Gregorio Marañón, Madrid, Spain; 2 Instituto de Investigación Sanitaria Gregorio Marañón, Madrid, Spain; 3 Medicine Department, School of Medicine, Universidad Complutense de Madrid (UCM), Madrid, Spain; 4 CIBER de Enfermedades Respiratorias (CIBERES CB06/06/0058), Madrid, Spain; 5 Instituto de Salud Carlos III, Madrid, Spain

## Abstract

**Objectives:**

To evaluate the clinical impact of a bedside visit to patients with a positive *Clostridioides difficile* test on the antimicrobial stewardship of *C. difficile* infection (CDI) and non-*C. difficile* infections.

**Methods:**

All patients ≥18 years old with positive CDI laboratory tests hospitalized between January 2017 and August 2017 received an immediate bedside intervention that consisted mainly of checking protective measures and providing recommendations on infection control and the management of CDI and other infections.

**Results:**

A total of 214 patients were evaluated. The infectious disease (ID) physician was the first to establish protective measures in 25.2% of the cases. In 22/29 (75.9%) cases, physicians in charge accepted ID consultant recommendations to stop CDI treatment in asymptomatic patients. Unnecessary non-CDI antibiotics were discontinued in 19.1% of the cases. ID recommendations were not accepted by physicians in charge in only 12.6% of the cases.

**Conclusions:**

A bedside rapid intervention for patients with a CDI-positive faecal sample was effective in avoiding overdiagnosis and unnecessary antibiotic treatment, optimizing anti-CDI drugs, increasing compliance with infection control measures and providing educational advice.

## Introduction

*Clostridioides* (*Clostridium*) *difficile* infection (CDI) is the most frequent cause of nosocomial diarrhoea and an important cause of morbidity and mortality in hospitalized patients.^1–4^ CDI usually occurs in severely ill patients after antibiotic prescription due to proven or suspected infections.^5–9^ CDI represents a significant burden on healthcare systems.^10–15^

Laboratory tests for the diagnosis of CDI are not always well interpreted by clinicians and the clinical situation is not easily assessed from the microbiology bench.^16–28^

There are several papers reporting the impact of an antibiotic stewardship intervention on the incidence of CDI.^29–31^ In contrast, we have just found one paper describing the role that a timely and early consultation by an infectious disease (ID) specialist for patients with a positive *C. difficile* laboratory test can play in improving adherence to clinical practice guidelines for the management of CDI^32^ and two other papers where pharmacists implemented a programme of antimicrobial stewardship, but did not play a role in the management of CDI.^33^^,^^34^ On the other hand, Rock *et al.*^35^ commented on the importance of proper diagnosis of cases with CDI but did not focus on the management or treatment of such patients. Moreover, Hecker *et al.*^36^ described a stewardship programme but focused only on patients who had previously received a faecal microbiota transplant.

Harpe *et al.*^37^ analysed patients with CDI who continued to receive antibacterial agents after their CDI diagnosis compared with patients who did not continue therapy. Hospital length of stay, mortality and subsequent admissions among patients who continued their antibacterial therapy remained significantly higher after adjusting for confounding variables, suggesting an opportunity for antimicrobial stewardship programmes to make important contributions to patient care.

Our study consisted of the evaluation of the clinical impact of a bedside visit to all patients with a positive *C. difficile* test, immediately after the laboratory result, with input to the physicians, nurses and relatives, on the antimicrobial stewardship of CDI and other infections.

## Patients and methods

### Setting

Our institution is a large teaching general hospital with 1550 beds. The clinical microbiology laboratory receives samples from hospitalized and non-hospitalized patients.

### Design and study population

One ID physician was called immediately after any *C. difficile* laboratory tests were returned as positive. This ID physician instituted an immediate bedside intervention, discussing the patient with the attending physician and including this as a standard ID consultation in the patient chart. Only hospitalized patients were included. This prospective study was conducted between January 2017 and August 2017.

### Laboratory procedures

For each CDI episode only one sample was considered. Rapid tests were performed on all samples with a clinical request for *C. difficile* diagnosis. The rapid detection test consisted of a two-step diagnostic algorithm based on a first immunochromatographic antigen detection of glutamate dehydrogenase (GDH) and toxins A/B (direct toxin test) simultaneously (C. DIFF QUIK CHEK COMPLETE assay, TechLab, Blacksburg, VA, USA) and secondly, samples with either or both of the previous tests positive were tested by a real-time PCR for the B toxin gene (Xpert^™^*C. difficile* assay, GeneXpert, Cepheid, Sunnyvale, CA, USA).

Furthermore, all samples were also tested by toxigenic culture (TC).^19^ TC was performed on *Clostridium* selective agar medium (bioMérieux) and plates were incubated under anaerobic conditions at 35°C–37°C for 48 h. Following incubation, colony morphotypes compatible with *C. difficile* were selected with the help of a binocular magnifying glass if necessary. Identification of colonies suspected of being toxigenic *C. difficile* (TCD) was confirmed using the immunochromatographic system previously described (C. DIFF QUIK CHEK COMPLETE assay).

There were certain patients diagnosed with CDI who had a negative direct toxin test but subsequently had a positive PCR or a positive TC. There were other patients who had a positive direct toxin test that also had a positive PCR and a positive TC.

### Definitions

A confirmed episode of CDI was defined as the presence of a positive result for toxigenic *C. difficile* testing in a patient suffering from diarrhoea (≥3 unformed stools in 24 h) or other abdominal symptoms, such as paralytic ileus, following the ESCMID recommendations.^38^^,^^39^

Severity of CDI was defined according to the guidelines of IDSA and the Society of Healthcare Epidemiology of America (SHEA).^40^

A recurrence was considered to have occurred when, after recovery from a previous episode (at least 3 days without diarrhoea and clinical improvement), symptoms returned and a stool sample separated from the former by between 15 and 60 days proved to be positive.

Death was considered to be CDI related when occurring within 10 days of the CDI diagnosis due to well-known complications of CDI.

### Bedside intervention

A single ID physician performed an unrequested bedside visit that was always accepted by the physician in charge. Recommendations were made as follows:

- Whether to establish or discontinue protective measures.
- Infection control and prevention, for patients, relatives, doctors, nurses and other clinical staff.
- Diagnostic process was explained to the physician, based on laboratory findings and clinical symptoms.
- Treatment for the management of CDI (Table 1).
- Whether other antimicrobials not devoted to CDI should be changed, removed or continued, according to local protocols.
- Whether different diagnostic tests, related or unrelated to CDI, should be performed.

**Table 1.** CDI treatment protocol

Circumstances	Treatment protocol
Initial episode (non-severe)	metronidazole 500 mg q8h for 10 days
Initial episode (severe)	vancomycin 125 mg q6h for 10 days
Ribotype 027	vancomycin tapering/pulsed
Initial episode (fulminant)	IV metronidazole 500 mg q8h for 10 days plus vancomycin retention enema 500 mg/100 mL saline q6h or combined with vancomycin 125 mg q6h for 10 days by oral/nasogastric tube
Recurrences	fidaxomicin 200 mg q12h for 10 days or
	vancomycin tapering/pulsed or
	vancomycin plus faecal microbiota transplant

The data collected included age, sex and hospital department, clinical data on the severity of the CDI episode and outcomes (treatment failure, recurrence, mortality and CDI-related mortality).

We performed a brief economic analysis, measuring the hours the ID physician and the other staff dedicated to evaluate every patient; we calculated the savings due to the reduction of days of antimicrobials (estimating that the reduction of days of metronidazole or vancomycin was around 9 days and of the rest of the antimicrobials around 7 days per episode).

### Data analysis

Data were analysed using STATA Version 12.0. Qualitative variables appear with their frequency distribution. Quantitative variables are expressed as the median and IQR. Proportions were compared using the Fisher exact test (two-tailed). A *P* value of <0.05 was considered statistically significant.

### Ethics

As the study was based on routine clinical interventions, the local ethics committees approved the study and waived the requirement to obtain informed consent (MICRO.HGUGM.2019-021).

## Results

**Figure 1.**
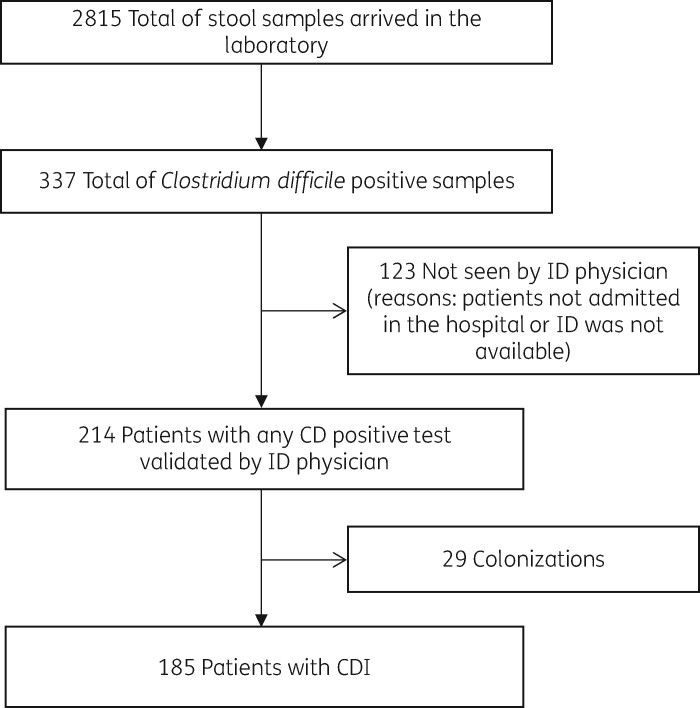
Study flow chart.

During the study period, a total of 2815 stool samples from 2027 patients aged over 18 years were sent for *C. difficile* diagnosis in our institution. Of these, 337 patients were diagnosed with a CDI, although only 214 (63.5%) of them could be evaluated at the bedside by an ID physician (Figure 1). Bedside intervention was accepted by physicians in charge and performed in all cases. Patients’ median age was 74 years and 95 patients (44.4%) were male.

The distribution of our cases, according to the potential place of acquisition was as follows: hospital-onset healthcare-facility-associated (HO-HCFA) CDI accounted for 172 (80.4%) cases; community-onset healthcare-facility-associated (CO-HCFA) CDI accounted for 29 (13.6%); 9 (4.2%) were hospitalized community-acquired episodes and 4 (1.9%) were indeterminate.

Overall, 194 (90.7%) were first episodes and 20 (9.3%) were recurrences. Most patients (162, 75.7%) were admitted to medical wards. Regarding clinical presentation, CDI cases were mild in 107 (50.0%), severe in 75 (35.0%) and severe-complicated in 3 (1.4%). *C. difficile* ribotype 027 was detected in 15 (7.0%) cases, presenting as severe in 5 cases (33.3%).

Regarding protective isolation, the ID physician was the first to be aware of the laboratory test result and established protective measures, that had not yet been taken, in 54 (25.2%) cases. The remaining patients were already on preventive protective measures.

Overall, 108 (50.5%) physicians, 141 (65.9%) nurses and 199 (93.0%) patient companions required and received additional prevention advice.

CDI treatment was initiated at the recommendation of the ID physician at the time of the visit in 76 (35.5%) patients.

The ID physician was instrumental in identifying 29 cases (13.6%) as only colonized with *C. difficile*, in which faecal sample collection was unnecessary, defined as fewer than three diarrhoeic stools within 24 h or after patient recovery. In 22 of these 29 cases (75.9%), physicians in charge agreed to stop or not initiate CDI treatment following the ID physician’s recommendations.

At the time of the study, the most common initial medication was metronidazole, in 113 patients (52.8%), followed by vancomycin in 91 (42.5%) and fidaxomicin in 10 (4.7%). Treatment changes were recommended in 48 (22.4%); 6 (12.5%) were switched from IV to oral metronidazole, 36 (75.0%) were upscaled from metronidazole to vancomycin and 6 (12.5%) from vancomycin to fidaxomicin. A microbiota transplant was recommended and performed in 8 (3.7%) cases.

Regarding the non-CDI-oriented antimicrobial treatment of the patients, antibiotics were discontinued in 41 (19.2%) cases. Other ID interventions included the recommendation of other diagnostic tests to complete patient management in 38 (17.8%) cases.

Laboratory tests were divided as follows: 96 (44.9%) of the population had direct detection of toxin in stool. The remaining 118 (55.1%) were negative for direct detection of toxin but had a positive TC with or without positive PCR test (nucleic acid amplification test; NAAT). These cases are compared in Table 2.

**Table 2.** Intervention for patients diagnosed by a positive or negative direct toxin test

	Direct toxin test result			
Characteristic	positive (*n *=* *96)	negative (*n *=* *118)	Total (*n *=* *214)	*P* value
Colonization	11 (11.5%)	18 (15.3%)	29 (13.6%)	0.548
CDI treatment initiated by ID physician	34 (35.4%)	42 (35.6%)	76 (35.5%)	1
Stopped CDI treatment	17 (17.7%)	24 (20.3%)	41 (19.2%)	0.728
Treatment changes	25 (26.0%)	23 (19.5%)	48 (22.4%)	0.323
Recommendation of another diagnostic test	15 (15.6%)	23 (19.5%)	38 (17.8%)	0.479
Recommendations not followed	10 (10.4%)	17 (1.4%)	27 (12.6%)	0.415

There were no significant differences in the type of intervention required between direct toxin-positive or direct toxin-negative patients.

Death occurred in 51 patients (23.9%) during the period of admission, but was clearly related to CDI in only 3 patients (1.4%).

The calculated cost of the ID physician intervention in the 214 patients (salary), considering the ID time payment, was estimated to be €6800 during the study period, while the estimated cost of the savings from antimicrobial discontinuations only were estimated at €1799.

ID recommendations were not accepted by physicians in charge in 27 (12.6%) cases.

## Discussion

Our work shows the impact of a timely intervention by a single ID physician in patients with one or more positive laboratory tests for CDI, mainly on the reduction of unnecessary antimicrobials, both for CDI and for other systemic infections.

The incidence of CDI seems to be increasing in many institutions and also in the community. CDI is currently, at least numerically, the most frequent infection in hospitalized patients.^7^^,^^9^^,^^41–43^

Regardless of the cost in morbidity and mortality, the economic expense caused by CDI is estimated at more than €6000 per episode in most of the studies that have evaluated this problem.^13^^,^^44^^,^^45^

Despite the low strength of evidence in the reviewed studies, the consistency of the findings suggests a positive impact of antimicrobial stewardship programmes on the prevention and control of nosocomial CDI.^46–50^

However, information regarding intervention in CDI cases is very scarce. Fabre *et al.*^51^ reviewed the charts of adult patients with positive CDI tests to evaluate clinical practices and generate management recommendations provided by a CDI working team, after case discussions and education at the Johns Hopkins Hospital in Baltimore. In their study, recommendations were required in a high proportion of patients (84 of 96 cases) and providers accepted 43% of CDI recommendations. They were also able to improve antibiotic selection for non-*C. difficile* infections. The authors selected patients with positive NAATs alone. Our results are concordant with the former study but our series is larger, we included patients with any *C. difficile* toxigenic positive test and not NAAT-positive cases only, the intervention was provided by a single ID physician and our proportion of acceptance of recommendations was much higher. Avoidance of treatment in patients who were only colonized was one of the main interventions but a high proportion of modifications in CDI and non-CDI treatment was also achieved.

We have only been able to make a very simple economic analysis. Data on the reduction of costs for the use of antibiotics is very scarce, but it appears likely that the benefit of reducing indirect costs that we were not able to estimate is much greater.

Our study also shows the impact of the old guidelines, recommending metronidazole as the treatment of choice for patients with mild to moderate CDI, in contrast with the minimal use of metronidazole according to the most recent guidelines.^40^^,^^52^

The microbiology laboratory is the first to know about the presence of toxigenic *C. difficile* and the immediate intervention of an ID physician allows introduction of diarrhoea contact precautions. The large proportion of patients who do not meet the criteria for diarrhoea and are simply carriers makes it possible to withdraw treatment from a large contingent of patients.

The comparison between patients with and without positive direct toxin results shows that the intervention is useful in both types of patients (direct toxin positive or direct toxin negative).

The limitations of our study were as follows: first, it was performed in a single centre, so the number of patients is not sufficient to give robust clinical and economic results; and second, the descriptive nature of the study. We preferred to intervene across the whole institution rather than apply a case–control study comparing intervention versus no intervention.

In summary, a rapid bedside intervention on all CDI-positive faecal sample patients is effective in avoiding overdiagnosis and unnecessary antibiotic treatment, optimizing anti-CDI drugs, increasing compliance with infection control measures and providing educational advice.

## Funding

This study was partially financed by Instituto de Salud Carlos III (PI3/00687, PI16/00490, PIE16/00055).

## Transparency declarations

None to declare.

## Supplementary Material

dlaa037_Supplementary_Data
